# Regular Exercise Correlates With Enhanced Self‐Control and Prefrontal Function in Excessive Short‐Video Users

**DOI:** 10.1002/brb3.71552

**Published:** 2026-06-14

**Authors:** Yawei Li, Tian Feng

**Affiliations:** ^1^ Department of Sport Henan Sport University Zhengzhou China; ^2^ School of Physical Education and Sport Science Fujian Normal University Fuzhou China; ^3^ Department of Physical Education Henan Sport University Zhengzhou China; ^4^ School of Physical Education Henan University Kaifeng Henan China

**Keywords:** exercise, functional near‐infrared spectroscopy, inhibitory control, prefrontal cortex, self‐control, short‐video overuse

## Abstract

**Objective:**

This study investigated the associations between physical activity and short‐video viewing duration on self‐control behavior and prefrontal cortical activation in college students.

**Methods:**

Male college students (*N* = 124) with problematic short‐video use (scale score ≥ 39) were initially recruited. Based on objective daily viewing time, they were classified into low (LV) (2.16 ± 0.54 h/day), moderate (MV) (4.24 ± 0.67 h/day), and high (HV) (6.17 ± 0.87 h/day) usage groups. Based on exercise habits, participants were further divided into high (HE) (≥ 3 sessions/week, ≥ 1 h/session) and low (LE) (≤ 1 session/week) exercise groups. Fifty‐eight participants completed the 2 (exercise) × 3 (video usage) design. Inhibitory control was assessed using a Go/Nogo task, and functional near‐infrared spectroscopy (fNIRS) measured oxygenated hemoglobin changes across 20 prefrontal channels. Hierarchical regressions (continuous video duration) were the primary analysis; ANOVAs were used for descriptive visualization.

**Results:**

The HV group showed longer reaction times (RT) than the LV group, with no accuracy differences. The HE group exhibited shorter RT and higher self‐control and decisiveness scores than the LE group. A marginally significant interaction on decisiveness indicated that within MV and HV groups, HE individuals scored higher than LE individuals. Neurally, the HV group showed greater activation in right dorsolateral and right ventrolateral prefrontal cortex than MV and LV groups. The LE group exhibited broader hyperactivation in left and right dorsolateral prefrontal cortex and frontopolar area compared to the HE group.

**Conclusion:**

Excessive short‐video use is associated with poorer behavioral self‐control and reduced prefrontal efficiency, with hyperactivation in right hemisphere regions. Regular exercise is related to better self‐control performance and enhanced prefrontal efficiency, particularly in MV and HV video users. These findings provide neurocognitive evidence linking exercise to reduced adverse effects of excessive digital media use.

## Graphical abstract

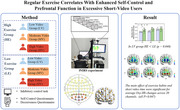

## 1 Introduction

Smartphone short‐video applications, with their algorithmic recommendations and fragmented presentation, have become the dominant form of digital entertainment, yet they have also precipitated widespread excessive use (Ye et al. 2023). According to official statistics, over 90% of online video users in China engage with mobile short videos, rendering “phone dependency” a pervasive societal norm (Chen et al. 2021; China Internet Network Information Center 2024). From the perspective of uses and gratifications theory, while short videos satisfy users' immediate psychological needs, they readily induce patterns of overuse (Katz et al. 1974). This excessive engagement is closely associated with emotional dysregulation, distorted time perception, and a range of adverse psychological and physiological consequences (Hasan et al. 2018; Ye et al. 2023). According to information overload theory, sustained exposure to excessive, high‐velocity streams of short‐video content depletes cognitive resources and impairs processing efficiency, thereby compromising higher order cognitive functions (Lee et al. 2016). Consequently, identifying effective strategies to mitigate excessive short‐video use and its associated cognitive impairments has emerged as a pressing societal priority.

Existing research indicates that the core psychological mechanism underlying excessive mobile short‐video use lies in deficient self‐control (Zhang et al. 2019). Self‐control refers to an individual's capacity to actively regulate behavior and make rational decisions when confronting temptations or impulses (Buzzell et al. 2020). Individuals with high self‐control more effectively curb excessive phone use and reduce unnecessary cognitive resource depletion (Lepp et al. 2013). This suggests that self‐control represents a key psychological construct for understanding excessive short‐video consumption. For many studies, exercise was demonstrated to be related to self‐control. Experimental research has shown that both 8‐week and 12‐week structured exercise programs significantly improve individuals' self‐control and decision‐making abilities (Gillebaart and de Ridder 2015; Milajerdi et al. 2020; Tse et al. 2019). The underlying mechanism may be partially explained by the physiological arousal hypothesis, which posits that exercise optimizes the availability and allocation efficiency of cognitive resources through physiological processes such as increased cerebral blood flow (Zhang and Liu 2019). However, a critical question remains unresolved: whether regular exercise habits are associated with better self‐control and prefrontal function in individuals with excessive short‐video use. Currently, empirical studies directly examining the relationship between exercise habits and excessive short‐video use remain scarce.

To fully elucidate the potential benefits of exercise and their underlying mechanisms, investigation at the cognitive neural level is essential. The prefrontal cortex is a core brain region regulating self‐control (Roberts and Wallis 2000), and studies have confirmed that exercise is associated with enhanced prefrontal activation (Cai et al. 2024). For instance, acute moderate‐intensity exercise improves activation in the left dorsolateral prefrontal cortex (DLPFC) while simultaneously enhancing inhibitory control task performance (Best 2010). In this context, functional near‐infrared spectroscopy (fNIRS) technology provides an ideal tool for synchronously monitoring prefrontal brain activity during cognitive task execution. This technique offers excellent motion tolerance, enabling continuous monitoring of cortical hemodynamic responses with minimal participant interference, making it particularly suitable for recording neural activity during cognitive tasks involving rapid responses or inhibitory control (Erfani and Abedin 2018; Meng et al. 2022). Emerging evidence indicates that hemodynamic indicators measured by fNIRS sensitively reflect cognitive control‐related brain functional changes and are closely associated with prefrontal activity (Herold et al. 2020). Nevertheless, a notable integrative gap persists in the current literature as few studies have incorporated habitual exercise levels, inhibitory control in populations with excessive short‐video use, and task‐synchronized fNIRS prefrontal neural monitoring within a single experimental framework. This lack of integration limits our understanding of whether exercise habits are associated with the core cognitive functions of individuals with excessive short‐video use.

Therefore, to address the aforementioned research gaps, the present study employed an integrated behavioral–neural experimental approach with college students exhibiting varying degrees of short‐video overuse. Specifically, we investigated the following questions: (1) Are habitual exercise levels associated with better behavioral performance of excessive short‐video users on inhibitory control tasks? (2) Are there differences in task‐induced activation patterns of key brain regions such as the DLPFC between high (HE) and low exercise (LE) groups? (3) Is there an association between inhibitory control behavioral performance and prefrontal neural activation across individuals with different exercise habits? By addressing these questions, this study aims to provide neurocognitive evidence regarding the relationships between exercise habits, self‐control, and prefrontal function in individuals with mobile short‐video overuse. The anticipated findings will not only deepen our understanding of the neurocognitive correlates underlying exercise benefits but also offer empirical support for developing lifestyle‐based strategies targeting behavioral health issues in the digital era.

## 2 Methods

### 2.1 Participants

This study initially recruited male college students. Inclusion criteria were (1) no history of cardiovascular disease or sports injuries and (2) no concurrent participation in other experiments affecting cognitive or neurological functions. To identify individuals with problematic short‐video use, we administered the Problematic Short Video Use Scale (Mao et al. 2022) via an online survey. This 13‐item scale uses a five‐point Likert scale, with total scores ≥ 39 indicating problematic use. In this sample, the scale demonstrated good internal consistency (Cronbach's *α* = 0.839). Participants were also classified by exercise habits based on objective records from health apps or exercise apps on their smartphones and wearable devices (e.g., smart bands). These records included weekly exercise frequency and duration per session over the past 3 months. Participants were categorized as HE (≥ 3 sessions/week, ≥ 1 h/session) or LE (≤ 1 session/week).

A total of 196 valid responses were collected (96 HE, 100 LE). After applying the cutoff score (≥ 39) and inclusion criteria, 124 eligible participants were identified, including 79 HE and 65 LE subjects. To obtain objective short‐video viewing time, we further collected participants' daily screen time data from their mobile devices over the past month. A total of 46 participants were excluded due to not having enabled app usage tracking or having incomplete records for less than 1 month, leaving 78 participants with valid objective viewing time data.

To further examine the interaction between cumulative short‐video viewing time and exercise habits, these 78 participants were divided based on their average daily short‐video screen time (hours/day, video playback only) over the past month, and Kelley's extreme grouping method (Kelley 1939) was used to categorize participants into three groups: the top 27% (daily viewing time > 5.00 h) were classified as the high video (HV) group (6.17 ± 0.87 h/day), the bottom 27% (daily viewing time < 3.14 h) as the low video (LV) group (2.16 ± 0.54 h/day), and the middle 46% as the moderate video (MV) group (4.24 ± 0.67 h/day). It is important to note that while this grouping was used for experimental recruitment and bar chart visualization, all primary statistical analyses (hierarchical regression) treated short‐video duration as a continuous variable.

This study employed a 2 (exercise: high, low) × 3 (video usage: high, moderate, low) between‐subjects factorial design. The initial stratified grouping yielded uneven subgroup sizes: HE‐LV (*n* = 19), HE‐MV (*n* = 15), HE‐HV (*n* = 10), LE‐LV (*n* = 16), LE‐MV (*n* = 9), and LE‐HV (*n* = 9). To ensure balanced sample sizes across the six groups, participants in groups with more than 10 individuals were randomly selected using SPSS random sampling to achieve approximately equal group sizes. A total of 58 participants completed all experimental procedures, forming the six groups (Table 1).  G*Power 3.1 analysis indicated adequate post‐hoc power (0.82) with the current sample size (*N* = 58, effect size *f* = 0.4, *α* = 0.05). One‐way ANOVA confirmed no significant age differences across groups, *F*(5, 52) = 0.323, *p* > 0.05. Independent‐samples *t*‐tests revealed no significant differences between the included (*n* = 58) and excluded (*n* = 66) participants in age, *t*(122) = 0.45, *p* = 0.653; self‐control scores, *t*(122) = 0.67, *p* = 0.504; or daily short‐video duration, *t*(122) = 0.38, *p* = 0.705, suggesting that selection bias is unlikely to have substantially affected the results. All participants provided written informed consent before the experiment. This study was approved by the University Human Research Ethics Committee (approval no. 2024001).

**TABLE 1:** Participant information.

	Group	Number/people
High exercise group (HE)	Low video group (LV)	10
	Moderate video group (MV)	10
	High video group (HV)	10
Low exercise group (LE)	Low video group (LV)	10
	Moderate video group (MV)	9
	High video group (HV)	9

### 2.2 Materials

Inhibitory control task: Self‐control was assessed using a classic computerized Go/Nogo paradigm. Stimuli were the digits “2” (Go, 50% probability) and “8” (Nogo, 50% probability). Participants were instructed to press the left mouse button as quickly and accurately as possible for Go stimuli and to withhold responses for Nogo stimuli. Each stimulus was presented for 500 ms, with a randomly varying interstimulus interval of 800–1200 ms. The task comprised three blocks, each containing 50 trials (25 Go, 25 Nogo), with a 30‐s rest between blocks. Before the formal task, participants completed 10 practice trials to ensure task comprehension. Stimulus presentation and behavioral data recording (reaction time [RT] and accuracy) were controlled by E‐Prime 3.0.

Self‐control questionnaire: Self‐control was assessed using the Chinese version of the Self‐Control Scale, originally developed by Tangney et al. (2004) and revised by Tan and Guo (2008). This 19‐item scale uses a five‐point Likert scale (1 = “completely disagree” to 5 = “strongly agree”), with higher total scores indicating greater self‐control. The scale demonstrated good internal consistency in this sample (Cronbach's *α* = 0.84).

Decisiveness questionnaire: Decisiveness was measured using the decisiveness subscale of the Need for Cognitive Closure Scale (Liu and Liang 2007), revised by Liu and Liang (2007). This subscale consists of seven items rated on a six‐point scale and showed good reliability in this study (Cronbach's *α* = 0.84). Given prior evidence linking exercise to enhanced decisiveness (Milajerdi et al. 2020; Tse et al. 2019), and the unknown relationship between short‐video viewing and decisiveness, this measure was included to explore potential effects of both factors.

### 2.3 Procedure

**FIGURE 1:**
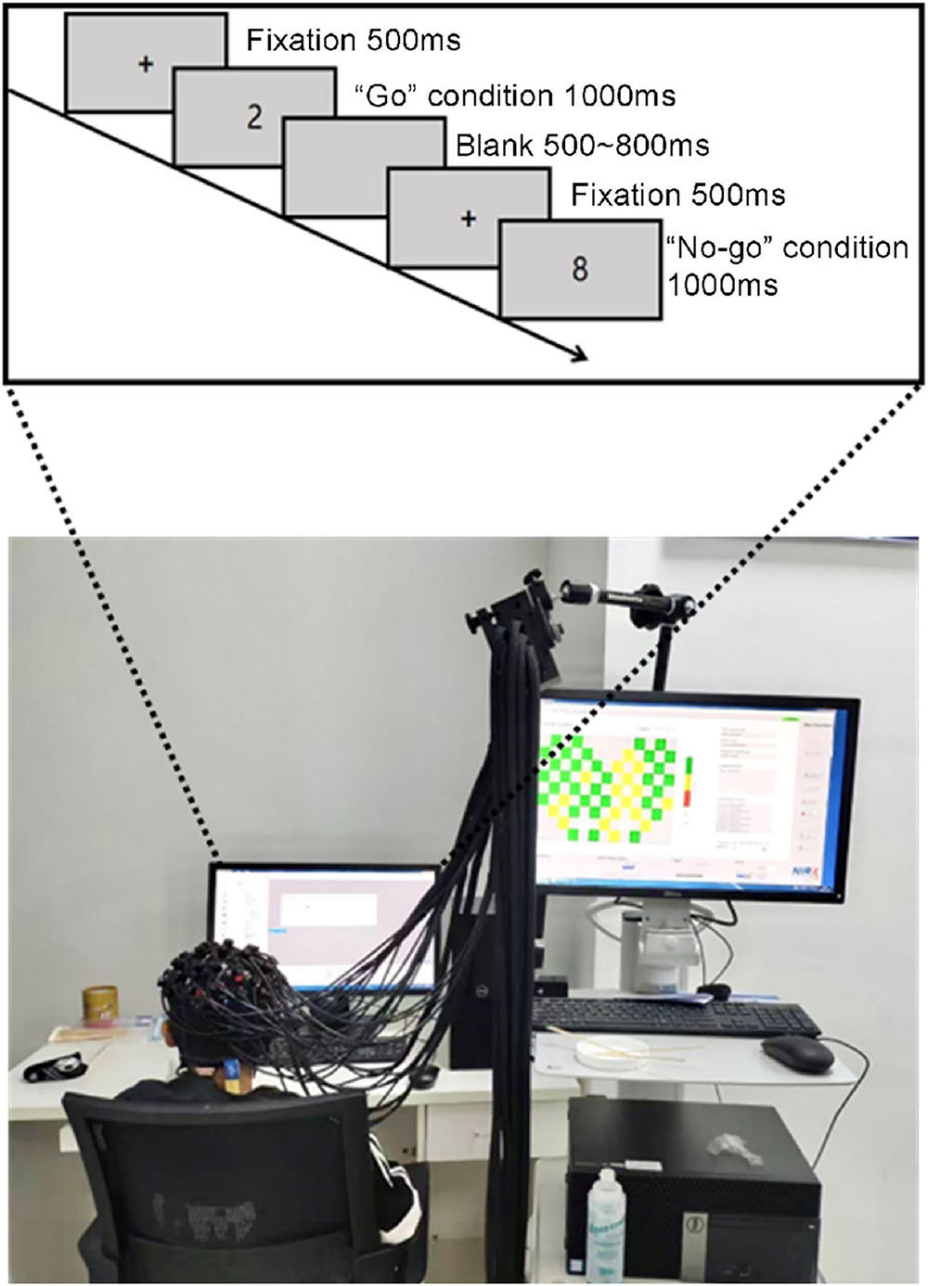
Experimental process.

All experiments were conducted individually in a sound‐attenuated, light‐controlled room (Figure 1). Upon arrival, participants provided written informed consent. The experimenter then fitted the participant with the fNIRS optode cap and verified signal quality for all channels. Participants were seated approximately 80 cm from the monitor. The procedure consisted of two phases: (1) Resting‐state recording: Participants rested quietly for 3 min while fixating on a central cross, providing baseline data for subsequent correction of task‐related signals. (2) Task execution: After reviewing task instructions, participants completed the practice trials followed by the three blocks of the Go/Nogo task. Throughout the experiment, the experimenter monitored fNIRS signal quality in real time and recorded any anomalies.

### 2.4 Data Acquisition

**FIGURE 2:**
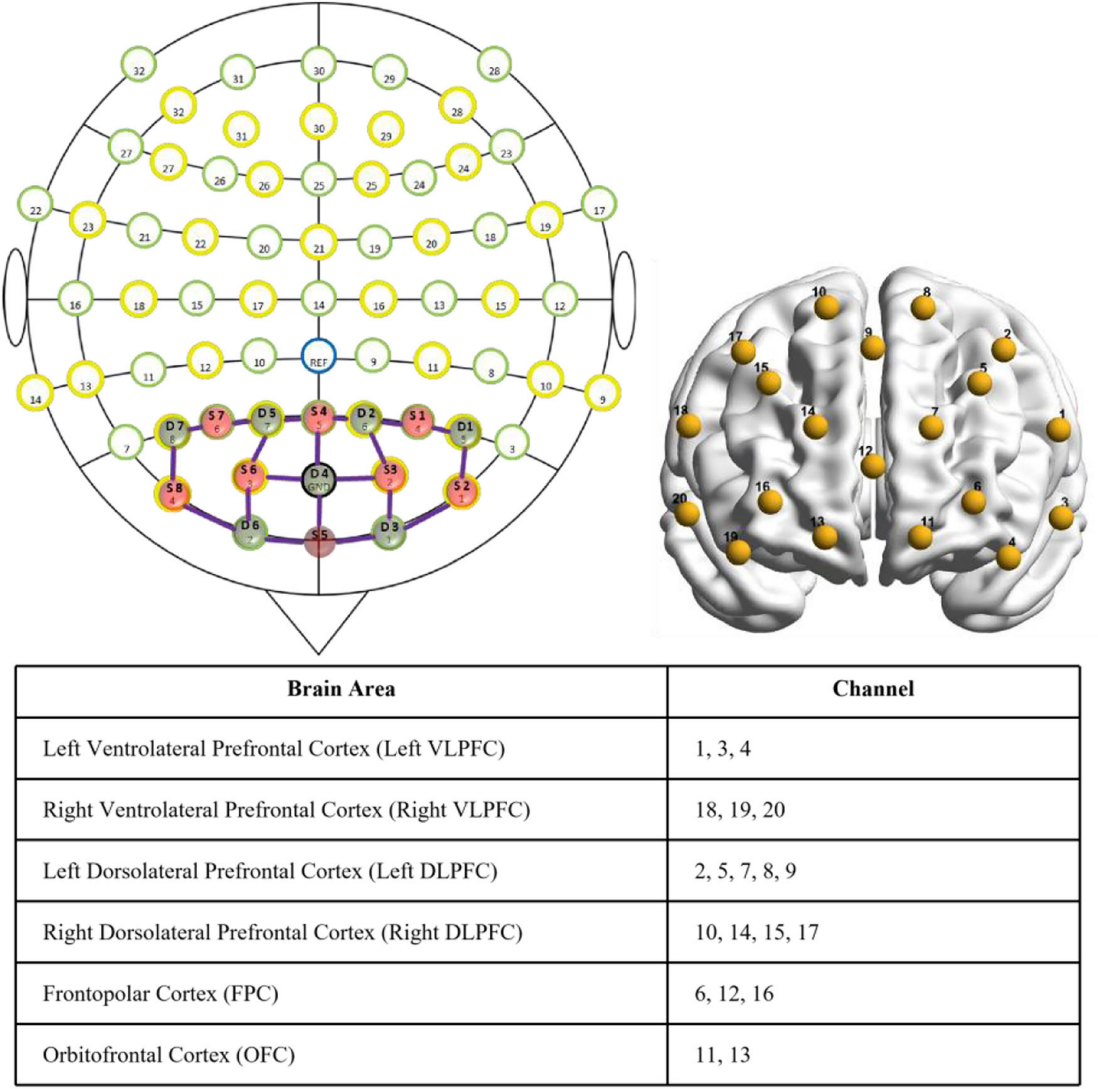
Near‐infrared channel layout and corresponding Brodmann area distribution.

Behavioral data (RT for correct Go trials; accuracy for Go and Nogo trials) were recorded using E‐Prime 3.0. Hemodynamic signals from the prefrontal cortex were collected using the NIRScout system (NIRx Medical Technologies, USA), which employs dual‐wavelength (780 and 830 nm) light sources at a sampling rate of 10.0 Hz. Based on the international 10–20 system, eight sources and seven detectors were arranged, forming 20 measurement channels over the prefrontal region. Channel coordinates were registered to Montreal Neurological Institute (MNI) standard space using a 3D digitizer or standard reference points to identify corresponding anatomical regions (Figure 2). The optode cap ensured good probe–scalp contact, and hair was parted to minimize signal attenuation.

### 2.5 Data Analysis

Behavioral data: Behavioral data were analyzed using SPSS 23.0. All variables met normality assumptions (Kolmogorov–Smirnov test, *Z* > 1.018, *p* > 0.252) and are presented as mean ± standard deviation (M ± SD). To test whether exercise habits moderate the relationship between short‐video duration and self‐control, hierarchical regression analyses were conducted. Prior to analysis, short‐video duration was centered to reduce multicollinearity between the main effects and the interaction term (Aiken and West 1991). For each behavioral and fNIRS outcome, the following procedure was applied. In Step 1, centered short‐video duration and exercise habits (coded as 0 = LE, 1 = HE) were entered as main effects. In Step 2, the interaction term (centered short‐video duration × exercise habits) was added. The significance of the interaction was determined by the change in *R* ^2^ (Δ*R* ^2^) and the *t*‐test of the interaction term. Variance inflation factor (VIF) was examined to assess multicollinearity. To further illustrate differences across levels of short‐video duration, two‐way ANOVAs were conducted with exercise habits (high, low) and short‐video usage time (high, moderate, low) as independent variables, and Go RT, Nogo accuracy, and self‐control scale score as dependent variables.

fNIRS data: Analysis focused on oxygenated hemoglobin (OxyHb) due to its superior signal‐to‐noise ratio and sensitivity to task‐related neural activation (Izzetoglu et al. 2007). Preprocessing was performed using NirsLAB software, including (1) conversion of light intensity to optical density, (2) removal of poor‐quality channels, (3) bandpass filtering (0.01–0.2 Hz) to remove low‐frequency drift and high‐frequency physiological noise, (4) principal component analysis for motion artifact correction, and (5) calculation of hemoglobin concentration changes using the modified Beer–Lambert law.

For each trial, OxyHb signals were extracted from −2 to 20 s relative to Nogo stimulus onset. Signals were then averaged across all Nogo trials for each participant and each channel to derive task‐related hemodynamic response curves. Given the large number of channels (20), separate two‐way ANOVAs (exercise × video usage) were conducted on mean OxyHb concentrations for each channel during the 4–14 s poststimulus window. To address multiple comparisons, false discovery rate (FDR) correction was applied to the resulting *p*‐values. Channels with FDR‐corrected *p* < 0.05 were considered significant. For channels with significant main effects or interactions, post‐hoc comparisons were performed using Bonferroni correction.

## 3 Results

### 3.1 Inhibitory Control Task

**FIGURE 3:**
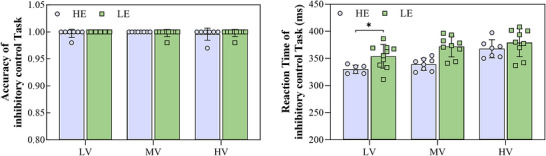
Behavioral results of the inhibitory control task across groups. (Left) Accuracy of inhibitory control task. (Right) Reaction times of inhibitory control task. Data are presented as individual data points (dots) with group mean ± SD. Simple effects analyses showed that within the low video group, the high exercise group exhibited significantly shorter reaction times on the task compared to the low exercise group (*p* = 0.040). HE, high exercise group; HV, high video group; LE, low exercise group; LV, low video group; MV, moderate video group.

Accuracy: Hierarchical multiple regression was conducted to examine the predictive effects of short‐video use time and exercise habits on inhibitory accuracy, as well as their interactive effect. In the first step, the main effects of short‐video use time and exercise habits were entered into the regression model. The results showed that short‐video use time could not significantly predict inhibitory accuracy (*β* = −0.129, *p* = 0.432), and the main effect of exercise habits was also nonsignificant (*β* = −0.099, *p* = 0.545). In the second step, the interaction term between short‐video use time and exercise habits was further included. The interaction effect did not reach statistical significance (*β* = −0.149, *p* = 0.521). In addition, a two‐way ANOVA was performed based on the extreme grouping of short‐video use time (low, moderate, high) and exercise habits. Consistent with the regression results, ANOVA showed no significant main effect of short‐video use time, *F*(2, 51) = 0.293, *p* = 0.747, *ηp* ^2^ = 0.008; no significant main effect of exercise habits, *F*(1, 51) = 0.029, *p* = 0.865, *ηp* ^2^​ = 0.001; and no significant interaction effect, *F*(2, 51) = 0.412, *p* = 0.665, *ηp* ^2^ = 0.016. The results of accuracy across all groups are presented in Figure 3.

RT: For Go trial RTs, short‐video use time significantly predicted RT (*β* = 0.424, *p* = 0.003), while the main effect of exercise habits was marginally significant (*β* = −0.255, *p* = 0.062) in Model 1 (Table 2). When the interaction term was entered in Model 2, the interactive effect of short‐video use time and exercise habits was nonsignificant (*β* = 0.153, *p* = 0.419). Moreover, the two‐way ANOVA based on short‐video extreme grouping further showed a significant main effect of short‐video time, *F*(2, 51) = 4.127, *p* = 0.022, *ηp* ^2^ = 0.137. Post‐hoc comparisons indicated that the LV group responded significantly faster than the MV and HV groups (*p* = 0.019). A significant main effect of exercise habits was also found, *F*(1, 51) = 5.264, *p* = 0.008, *ηp* ^2^ = 0.158, with the HE group exhibiting shorter RTs than the LE group. The interaction between exercise habits and short‐video time was not significant, *F*(2, 51) = 1.893, *p* = 0.161, *ηp* ^2^ = 0.069. Simple effects analysis further revealed that within the LV group, the HE group exhibited significantly shorter inhibition RTs than the LE group (*p* = 0.040). The RT across different groups is presented in Figure 3.

**TABLE 2:** Hierarchical regression results for reaction time.

Model	Predictor	*B*	SE	*β*	*t*	*p*	VIF
Model 1	(Constant)	364.07	4.06		89.62	< 0.001	
	Short‐video time	0.92	0.29	0.424	3.184	0.003	1.23
	Exercise habits	−12.61	6.60	−0.255	−1.912	0.062	1.23
*R* ^2^ = 0.180, *F*(2, 52) = 5.70, *p* = 0.006							
Model 2	(Constant)	365.08	4.26		85.65	< 0.001	
	Short‐video time	0.67	0.42	0.309	1.589	0.119	2.61
	Exercise habits	−12.32	6.63	−0.249	−1.858	0.070	1.24
	Short‐video duration × exercise habits	0.47	0.58	0.153	0.815	0.419	2.44
*R* ^2^ = 0.188, Δ*R* ^2^ = 0.008, Δ*F*(1, 51) = 0.67, *p* = 0.419							

### 3.2 Self‐Control and Decisiveness Score

Hierarchical multiple regression was performed to examine the predictive effects of short‐video time and exercise habits on self‐control scores. Detailed hierarchical regression results are presented in Table 3. In Model 1, short‐video usage time significantly negatively predicted self‐control (*β* = −0.323, *p* = 0.009), and exercise habits significantly positively predicted self‐control (*β* = 0.343, *p* = 0.006). In Model 2, the interaction term was further entered, and the interactive effect between short‐video usage time and exercise habits was nonsignificant (*β* = 0.279, *p* = 0.168). Additionally, two‐way ANOVA revealed significant main effects of short‐video time on self‐control, *F*(2, 51) = 3.892, *p* = 0.026, *ηp*2​ = 0.132. Post‐hoc comparisons showed that the LV group scored significantly higher than both the MV and HV groups, and the MV group scored significantly higher than the HV group (all *p* < 0.05). A significant main effect of exercise habits was also observed for self‐control, *F*(1, 51) = 7.124, *p* = 0.010, *ηp* ^2^ = 0.123, with the HE group scoring significantly higher than the LE group. The interaction between exercise habits and short‐video time was not significant for self‐control, *F*(2, 51) = 1.892, *p* = 0.161, *ηp* ^2​^ = 0.069. Simple effects analysis indicated that within the LE group, the LV group scored significantly higher than the HV group (*p* = 0.027).

**TABLE 3:** Hierarchical regression analyses for behavioral and questionnaire outcomes.

Model	Predictor	*B*	SE	*β*	*t*	*p*	VIF
Self‐control							
Model 1	(Constant)	61.687	1.534		40.201	0.000	
	Short‐video time	−0.248	0.092	−0.323	−2.691	0.009	1.123
	Exercise habits	6.272	2.192	0.343	2.861	0.006	1.123
*R* ^2^ = 0.214, *F*(2, 52) = 7.07, *p* = 0.002							
Model 2	(Constant)	62.427	1.611		38.747	0.000	
	Short‐video time	−0.430	0.159	−0.560	−2.701	0.009	3.409
	Exercise habits	5.871	2.192	0.321	2.678	0.010	1.142
	Short‐video time × exercise habits	0.271	0.194	0.279	1.396	0.168	3.166
*R* ^2^ = 0.232, Δ*R* ^2^ = 0.018, Δ*F*(1, 51) = 1.95, *p* = 0.168							
Decisiveness							
Model 1	(Constant)	25.043	0.975		25.690	0.000	
	Short‐video time	−0.107	0.059	−0.244	−1.831	0.072	1.12
	Exercise habits	2.117	1.393	0.202	1.520	0.134	1.123
*R* ^2^ = 0.098, *F*(2, 52) = 2.82, *p* = 0.069							
Model 2	(Constant)	25.708	1.005		25.584	0.000	
	Short‐video time	−0.271	0.099	−0.616	−2.727	0.009	3.409
	Exercise habits	1.756	1.367	0.168	1.284	0.205	1.142
	Short‐video time × exercise habits	0.244	0.121	0.438	2.012	0.049	3.166
*R* ^2^ = 0.193, Δ*R* ^2^ = 0.095, Δ*F*(1, 51) = 4.05, *p* = 0.049							

**FIGURE 4:**
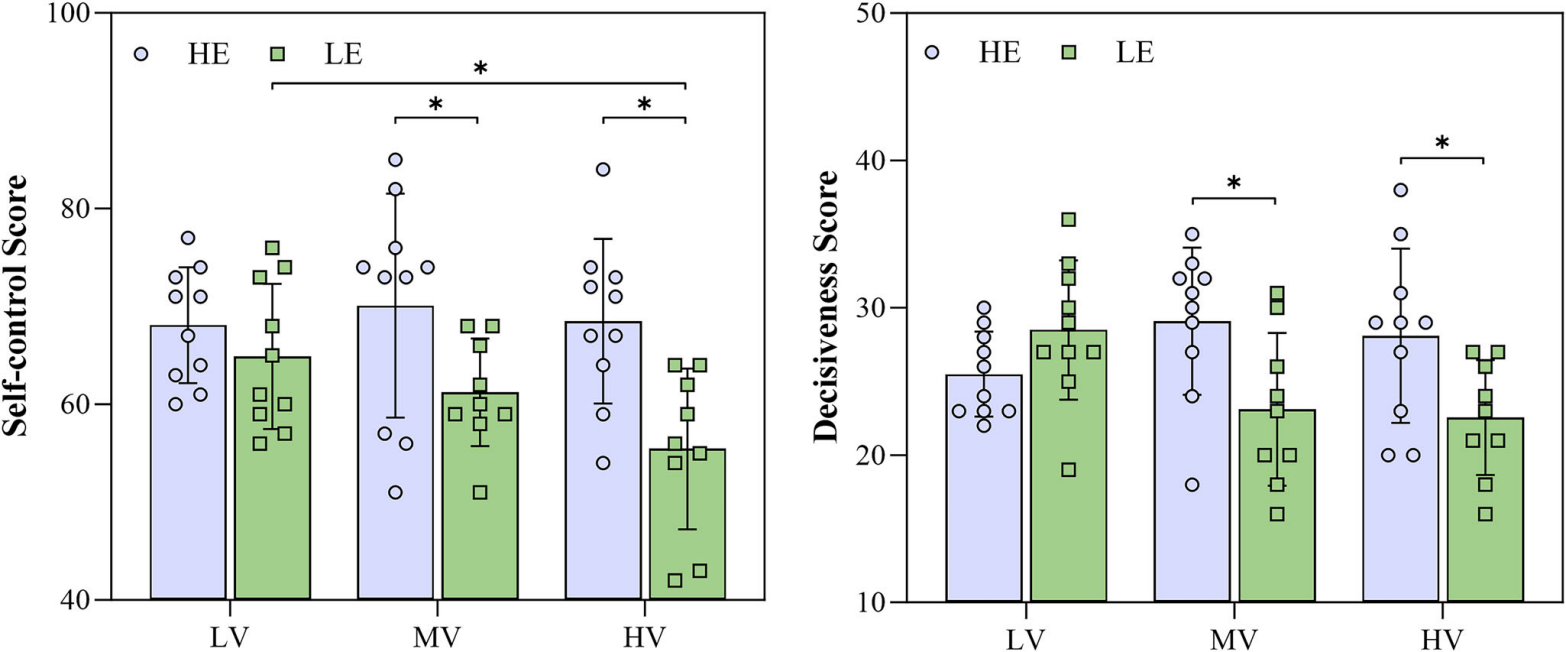
Self‐control and decisiveness scale scores across groups. (Left) Self‐Control Scale scores. (Right) Decisiveness Scale scores. Data are presented as individual data points (dots) with group mean ± SD. Simple effects analysis for decisiveness revealed that within the moderate and high video groups, participants in the high exercise group scored significantly higher than those in the low exercise group (*p* = 0.023 and *p* = 0.041, respectively). For self‐control, within the low exercise group, the low video group scored significantly higher than the high video group (*p* = 0.027). HE, high exercise group; HV, high video group; LE, low exercise group; LV, low video group; MV, moderate video group.

This study further examined the predictive patterns of short‐video use and physical exercise on decisiveness using hierarchical regression analysis. In the initial model, prolonged short‐video usage exhibited a marginally negative association with decisiveness (*β* = −0.244, *p* = 0.072), whereas exercise habits did not independently predict decisiveness (*β* = 0.202, *p* = 0.134). Notably, after incorporating the interaction term, a significant modulating interaction emerged between short‐video usage and exercise habits (*β* = 0.438, *p* = 0.049). Simple slope analysis revealed that for the LE group, short‐video duration had a significant negative effect on decisiveness, whereas for the HE group, the effect was not significant. Detailed regression results are shown in Table 3, and group comparisons are presented in Figure 4. ANOVA revealed significant main effects of short‐video time, *F*(2, 51) = 3.456, *p* = 0.039, *ηp*2​ = 0.119, and exercise habits, *F*(1, 51) = 6.532, *p* = 0.014, *ηp*2​ = 0.114. Overall decisiveness declined gradually with increasing short‐video exposure and was higher in the HE group. The two‐way interaction approached marginal significance in ANOVA (*p* = 0.058). Further simple‐effect decomposition confirmed that the protective effect of exercise was more prominent in MV and HV usage groups, where HE individuals displayed significantly higher decisiveness than their LE counterparts (*p* = 0.023, *p* = 0.041).

### 3.3 fNIRS

**FIGURE 5:**
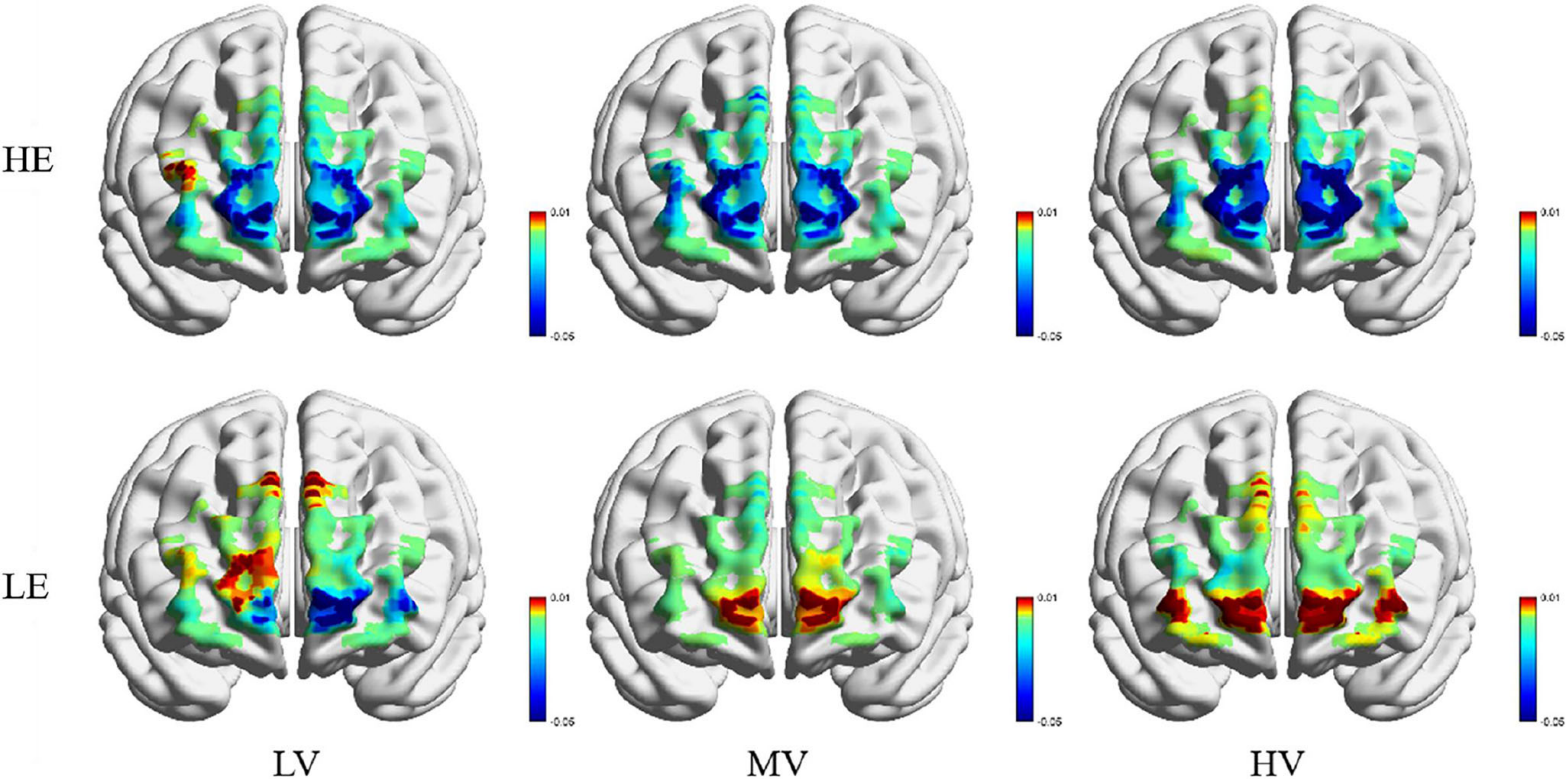
3D topographic maps of brain channel activation during the Go/Nogo task under experimental conditions. The color bar on the right represents the activation level, with redder colors indicating stronger activation and bluer colors indicating weaker activation. HS, high exercise group; HV, high video group; LE, low exercise group; LV, low video group; MV, moderate video group.

**FIGURE 6:**
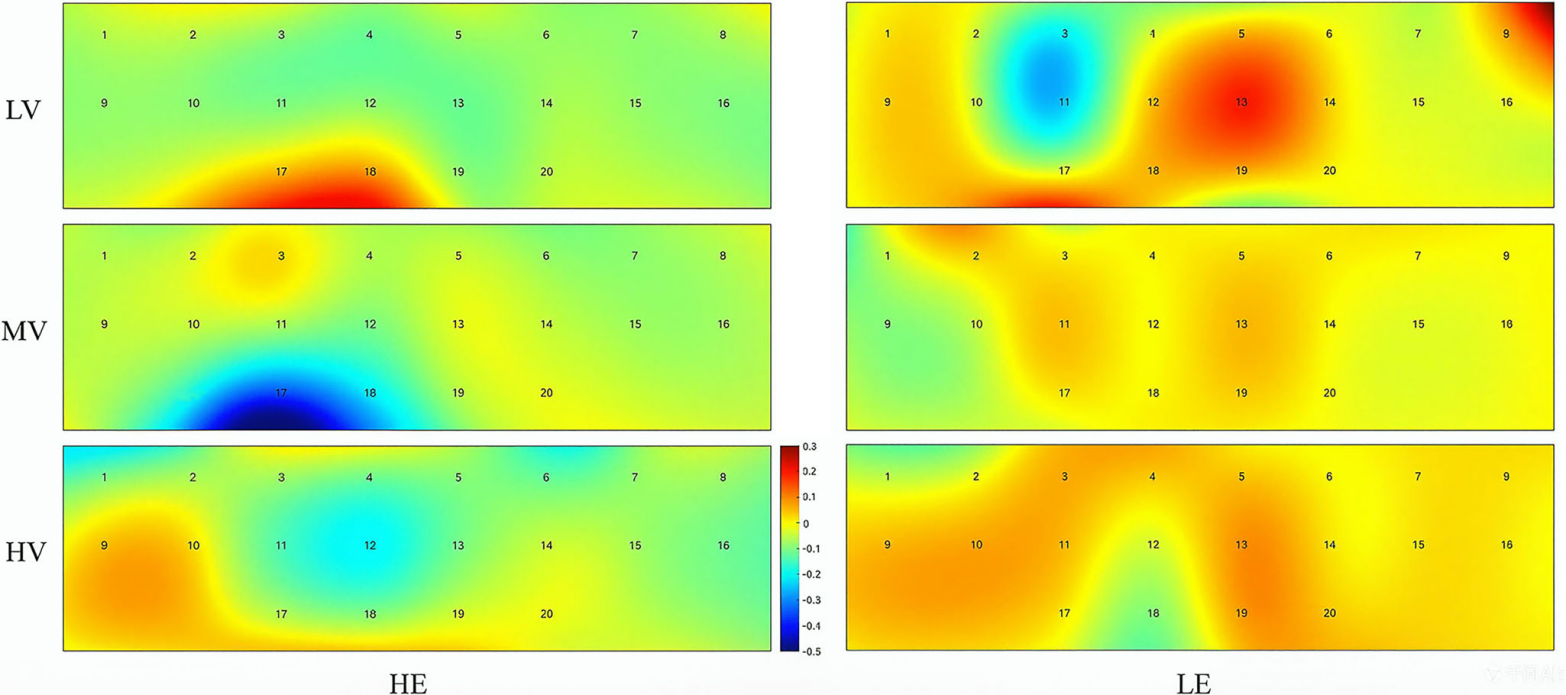
2D visualization of activation levels during the Go/Nogo task under experimental conditions. The color bar on the right represents the activation level, with redder colors indicating stronger activation and bluer colors indicating weaker activation. HE, high exercise group; HV, high video group; LE, low exercise group; LV, low video group; MV, moderate video group.

The topographic distribution of prefrontal cortex activation during the task is shown in Figures 5 and 6, visually illustrating the differences in activation patterns between groups.

Hierarchical regression analyses were performed for all 20 fNIRS channels. In Model 1, short‐video usage time exerted significant positive predictive effects on neural activation in CH4 (*β* = 0.270, *p* = 0.043), CH9 (*β* = 0.295, *p* = 0.035), CH10 (*β* = 0.369, *p* = 0.008), and CH19 (*β* = 0.307, *p* = 0.021). Regarding the main effect of exercise habits, significant negative predictions were found in CH6 (*β* = −0.439, *p* = 0.001), CH7 (*β* = −0.301, *p* = 0.031), CH12 (*β* = −0.301, *p* = 0.030), CH13 (*β* = −0.319, *p* = 0.022), CH14 (*β* = −0.301, *p* = 0.029), and CH15 (*β* = −0.276, *p* = 0.046). When the interaction term was added in Model 2, only CH12 presented a significant interactive effect between short‐video usage and exercise habits (*β* = 0.515, *p* = 0.022). Simple slope analysis revealed that for the LE group, short‐video duration had a significant negative effect on neural activation of CH12, whereas for the HE group, the effect was not significant. Detailed hierarchical regression coefficients across all fNIRS channels are summarized in Table 4.

**TABLE 4:** Hierarchical regression results for CH12.

Model	Predictor	*B*	SE	*β*	*t*	*p*	VIF
Model 1	(Constant)	−1.63 × 10^−^^6^	2.01 × 10^−^^5^		−0.081	0.936	
	Short‐video time	−3.42 × 10^−^^6^	1.72 × 10^−^^6^	−0.268	−1.988	0.052	1.12
	Exercise habits	−9.13 × 10^−^^5^	4.10 × 10^−^^5^	−0.301	−2.229	0.030	1.12
*R* ^2^ = 0.139, *F*(2, 52) = 4.20, *p* = 0.020							
Model 2	(Constant)	1.93 × 10^−^^6^	2.94 × 10^−^^5^		0.066	0.947	
	Short‐video time	−9.00 × 10^−^^6^	2.88 × 10^−^^6^	−0.705	−3.124	0.003	3.41
	Exercise habits	−1.03 × 10^−^^4^	3.96 × 10^−^^5^	−0.341	−2.610	0.012	1.14
	Short‐video time × exercise habits	8.32 × 10^−^^6^	3.52 × 10^−^^6^	0.515	2.365	0.022	3.17
*R* ^2^ = 0.197, Δ*R* ^2^ = 0.058, Δ*F*(1, 51) = 5.59, *p* = 0.022							

**FIGURE 7:**
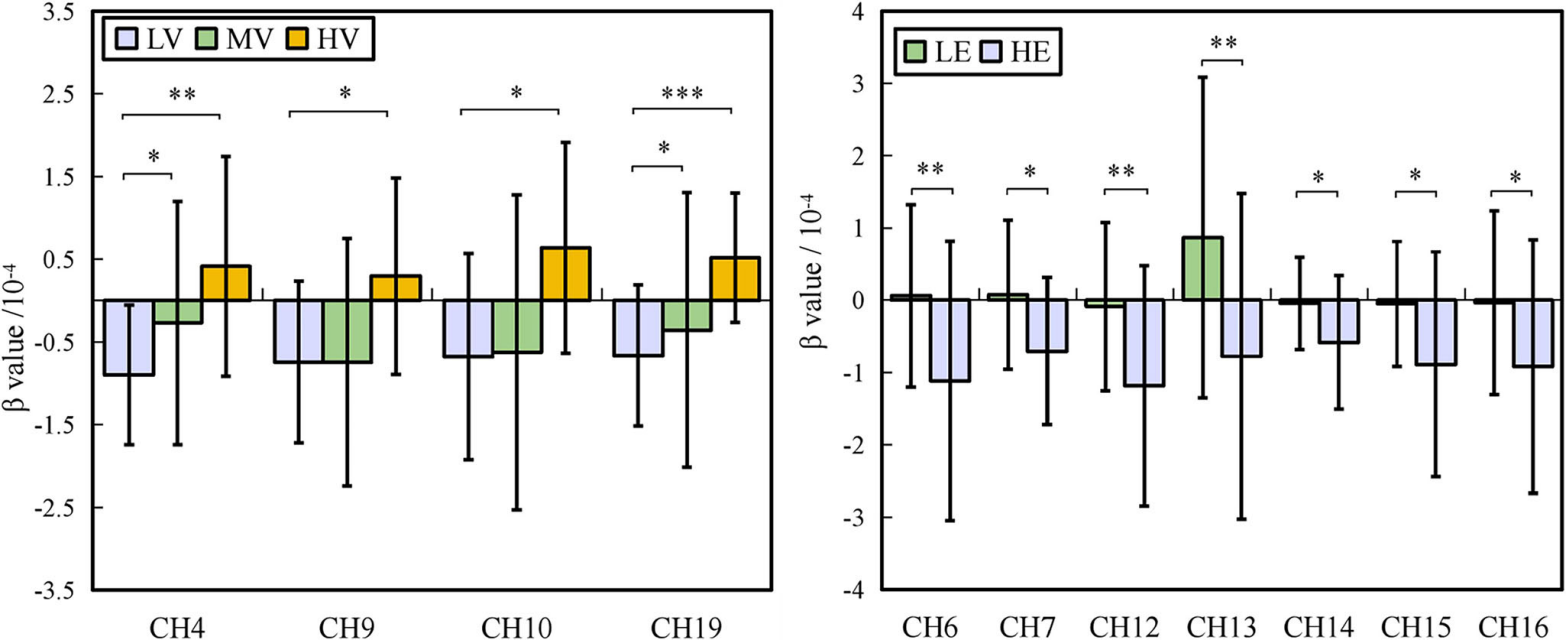
Average OxyHb changes in inhibitory control task under experimental conditions. (Left) *β* value for low, moderate, and high video groups. (Right) *β* value for low and high exercise groups. Data are presented as group bars with mean ± SD. A two‐way ANOVA was performed on the average OxyHb changes across 20 channels. The main effect of exercise habits and short‐video time were significant (all *p* < 0.047). HE, high exercise group; HV, high video group; LE, low exercise group; LV, low video group; MV, moderate video group.

To further demonstrate the differences across levels of short‐video time, a two‐way ANOVA was performed on the average OxyHb changes across 20 channels. All channel‐level *p*‐values were corrected for FDR to control for multiple comparisons. The main effect of exercise habits was significant in CH6, CH7, CH12, CH13, CH14, CH15, and CH16 (*p* < 0.047, *ηp* ^2^ > 0.075) (Figure 7). The LE group exhibited higher activation in the frontopolar region encompassing CH6, CH12, and CH16; the DLPFC including CH7, CH14, and CH15; and the orbitofrontal cortex where CH13 is located, compared to the HE group.

After FDR correction, the main effect of short‐video time was significant (*p* < 0.040, *ηp* ^2^ > 0.121) (Figure 7). Post‐hoc analysis revealed that the HV group exhibited higher activation levels in CH4 (left VLPFC), CH9 (left DLPFC), CH10 (right DLPFC), and CH19 (right VLPFC) compared to the MV and LV groups (*p* < 0.028). The MV group showed significantly higher activation in CH4 and CH19 than the LV group (*p* < 0.034). The interaction effect between exercise habits and short‐video time was not significant (all *p* > 0.05).

## 4 Discussion

### 4.1 Associations Between Exercise, Short‐Video Use, and Self‐Control Behavior

This study examined the associations between exercise and excessive short‐video use on individuals' self‐control behavior from both inhibitory control task performance and self‐report scales. The results revealed a dual pattern: Excessive short‐video use was associated with decreased self‐control, while regular exercise may exert a positive influence.

First, at the level of behavioral tasks, the impact of short‐video time on inhibitory control efficiency is mainly reflected in RT. The results show that the HV group had significantly longer RTs in the task compared to the LV group, but there was no significant difference in accuracy rates between the groups. Regression results confirmed this relationship, showing that short‐video time was a significant positive predictor of RT, indicating that longer short‐video usage was associated with slower inhibitory control responses. This finding suggests that excessive short‐video use may not impair an individual's basic ability to perform inhibitory control but significantly reduces their cognitive processing speed (Zhang et al. 2019). A possible explanation is that prolonged exposure to fragmented, highly rewarding short‐video information flows may lead to the continuous depletion of cognitive resources (such as attention and inhibitory control resources) (Baumeister et al. 2000), causing individuals to experience decreased processing efficiency or adopt a more cautious “trading speed for accuracy” strategy when faced with tasks requiring rapid responses. At the subjective experience level, the scale results are similar to the behavioral outcomes. Scores on the self‐control scale and the decisiveness subscale both indicate that higher short‐video time is associated with lower self‐control and decisiveness scores. Regression analyses further demonstrated significant negative associations between short‐video time and self‐control scores as well as decisiveness scores. This result aligns with the explanation of the “Uses and Gratifications Theory”: The immediate gratification provided by short videos may encourage users to extend their usage time (Katz et al. 1974), while excessive immersion could weaken their long‐term regulatory capacity over their own behaviors and decisions (Gillebaart and de Ridder 2015).

Second, in contrast to the aforementioned negative effects, regular exercise habits demonstrate a positive protective role. In the behavioral outcomes of inhibitory control, the HE group exhibited significantly shorter RTs compared to the LE group; in scale assessments, the HE group also scored significantly higher in self‐control and decisiveness. Regression analyses confirmed that exercise habits significantly positively predicted self‐control scores, and showed a marginally significant positive association with RTs (i.e., faster responses). This aligns with numerous studies concluding that physical exercise enhances cognitive functions (Best 2010; Brown and Bray 2018). This study further localizes this positive effect to the specific context of excessive short‐video use, suggesting that exercise habits may serve as an effective protective factor, associated with resistance to the self‐control depletion linked to excessive digital media consumption.

Finally, decisiveness is an extremely important component in the process of self‐control, representing an individual's ability to control themselves to quickly search for effective information and make unalterable decisions (Gillebaart and de Ridder 2015). Hierarchical regression analysis revealed a significant interaction between short‐video duration and exercise habits for decisiveness, indicating that the relationship between short‐video use and decisiveness differs between HE and LE groups. It should be noted that in the ANOVA, the interaction effect between exercise habits and short‐video duration on decisiveness was marginally significant. Given the exploratory nature of this finding and its potential theoretical relevance to understanding how lifestyle factors may interact to influence cognitive outcomes, post‐hoc simple effect analyses were conducted to further characterize the pattern of group differences. The results revealed that in the MV and HV groups, individuals in the HE group exhibited significantly better decisiveness than those in the LE group. This result suggests that for individuals who have developed moderate or high short‐video habits, regular exercise may be associated with preserved ability to make rapid and decisive decisions. Previous research has proposed that long‐term exercise is associated with better self‐control performance (Milajerdi et al. 2020). Together, these findings suggest that for individuals with moderate to high short‐video usage habits, regular exercise may be associated with preserved decisiveness abilities.

### 4.2 Associations Between Exercise, Short‐Video Use, and Prefrontal Activation

This study employed fNIRS to examine neurodynamic patterns associated with exercise habits and short‐video viewing duration during self‐control performance. Hierarchical regression analyses further quantified these relationships, providing more precise estimates of the associations between lifestyle factors and neural activation.

First, regarding the impact of short‐video intensity, this study identified a specific brain activation pattern: The HV group exhibited significantly higher activation in the right DLPFC and right VLPFC compared to the MV and LV groups, confirmed by the regression results showing significant positive associations between short‐video time and activation in CH4, CH9, CH10, and CH19. Extensive research has confirmed that activation of the right VLPFC is directly associated with the successful inhibition of inappropriate behaviors (Aron et al. 2004; Rubia et al. 2003). The right DLPFC plays a central role in conflict monitoring, task switching, and complex decision‐making (Barbey et al. 2013; Greene et al. 2001). Zimeo Morais et al. (2018) found that 95% of participants exhibited DLPFC activation during inhibitory control tasks. One interpretation of this activation pattern is that it may reflect greater cognitive effort or reduced processing efficiency among high‐frequency users. The behavioral finding of longer RTs without accuracy differences could be consistent with this view. An alternative possibility is that the heightened activation represents a successful compensatory mechanism that enables high‐frequency users to maintain adequate performance despite underlying challenges in processing speed. A third interpretation could involve individual differences in baseline arousal or motivation that were not measured in the current study. Prolonged exposure to high‐frequency, high‐reward short‐video content might affect the brain's excitation–inhibition balance, but this hypothesis requires direct testing in longitudinal or experimental designs.

Second, regarding the impact of exercise habits, this study observed that individuals in the LE group exhibited significantly higher activation intensities in multiple regions of the prefrontal cortex (including the left DLPFC, right DLPFC, frontopolar region, and orbitofrontal cortex) during inhibitory control tasks compared to the HE group. Regression analyses supported this pattern, showing significant negative associations between exercise habits and activation in CH6, CH7, CH12, CH13, CH14, and CH15, indicating that higher exercise levels were consistently associated with lower activation across these prefrontal regions. Unlike the pattern observed for short‐video duration, this activation pattern appeared more widespread rather than regionally specific. Combined with behavioral results showing shorter RTs and higher self‐control scores in the HE group, a plausible account is that the two groups differ in neural processing efficiency. According to the neural efficiency hypothesis, optimized and automated cognitive processing is typically accompanied by more focused and economical brain region activation (Klingberg 2010; Takeuchi et al. 2010). From this perspective, the LE group displayed stronger activation across functionally diverse brain regions: the DLPFC (working memory and advanced control), frontopolar region (multitask coordination and planning) (Koechlin and Hyafil 2007), and orbitofrontal cortex (reward evaluation and impulse control) (Burgess et al. 2007; Miller 2000). This may reflect less refined and automated cognitive control processes, that is, they may need to mobilize a broader neural network, exerting more conscious and effortful cognitive efforts to integrate information, evaluate outcomes, and execute inhibition, which can be viewed as an inefficient neural resource mobilization strategy (Cai et al. 2024). In contrast, the HE group's better behavioral performance accompanied by relatively more focused activation could be interpreted as evidence of higher neural efficiency, possibly resulting from exercise‐related enhancements in prefrontal blood flow, neuroplasticity, and network functional integration (Guida et al. 2012). This may enable individuals with HE habits to more rapidly and precisely recruit necessary neural resources during cognitive tasks, demonstrating higher neural efficiency—achieving superior cognitive output with less neural energy consumption. Therefore, exercise is associated with self‐control–related brain processing performance and activation patterns in individuals with excessive short‐video use. Notably, regression analyses revealed a significant interaction effect between short‐video time and exercise habits on CH12 activation. Simple slope analysis showed that for the LE group, short‐video time had a significant negative effect on CH12 activation, whereas for the HE group, the effect was not significant. This neural interaction pattern parallels the marginally significant behavioral interaction observed for decisiveness, suggesting that exercise habits may modulate the relationship between short‐video exposure and both prefrontal activation and cognitive performance.

In summary, the behavioral and neuroimaging findings of this study collectively point to a core mechanism—cognitive neural efficiency. Excessive short‐video use may be associated with a pattern of chronic cognitive load, which could be reflected in reduced processing efficiency of the prefrontal inhibitory control network (particularly the right‐hemisphere network). This might be accompanied by the need to mobilize additional, targeted resources to compensate for efficiency loss, behaviorally manifested as slowed RTs. In contrast, regular physical exercise is associated with patterns of brain health and plasticity, which may relate to more efficient functional integration and resource allocation within the prefrontal network, enabling individuals to achieve cognitive goals in a more economical and automated manner, behaviorally reflected in faster reactions and better self‐control. Thus, these two lifestyle factors show opposite associations with the same cognitive neural system.

This study has several limitations that should be acknowledged. While fNIRS offers high ecological validity for monitoring prefrontal activation during cognitive tasks, its detection range is limited to superficial cortical regions, preventing investigation of deep brain structures (e.g., basal ganglia, anterior cingulate cortex) and large‐scale whole‐brain networks. Second, to achieve balanced group sizes for the factorial design, participants in groups with more than 10 individuals were randomly subsampled. While this procedure facilitated balanced statistical comparisons, and the comparisons between included and excluded participants showed no significant differences in age, self‐control scores, or short‐video duration, it may reduce the representativeness of the sample. Third, the study employed a male‐only sample. This design choice was made to control for potential confounding effects of gender, as previous research has consistently documented significant gender differences in both self‐control abilities among Chinese students (Gaillard et al. 2020) and short‐video usage patterns among Chinese college students (Zhan and Zhu 2025). However, this also limits the generalizability of the findings beyond Chinese male college students with problematic short‐video use. Additionally, the cross‐sectional design of this study does not allow for causal inferences regarding the relationships between exercise, short‐video habits, and brain function. Future studies could employ multimodal techniques combining fNIRS and EEG to simultaneously assess hemodynamic and electrophysiological activity, or conduct longitudinal intervention studies to examine whether changes in exercise or short‐video habits are associated with corresponding changes in inhibitory control–related brain activation patterns. Future research should also include more diverse samples, including female participants, to explore potential gender differences in the relationships between exercise, short‐video exposure, and self‐control outcomes.

## 5 Conclusion

This study integrated behavioral measurements with fNIRS to examine the associations between exercise habits and short‐video viewing duration and self‐control ability and its neural correlates. At the behavioral level, this study identified significant negative associations between excessive short‐video use and self‐control ability, manifested by higher usage time being associated with slower inhibitory control RTs and lower self‐control scale scores. More importantly, regular exercise habits were associated with better inhibitory control behavioral performance and self‐reported self‐control levels. At the neural level, this study provides neurocognitive evidence regarding the relationships between exercise and short‐video behaviors and self‐control. fNIRS data showed that individuals with LE habits and high short‐video time exhibited broader and stronger activation in the prefrontal cortex (particularly the DLPFC and VLPFC) when performing inhibitory control tasks. One plausible interpretation of this pattern is that it may reflect relative neural inefficiency or compensatory hyperactivation: Individuals may need to mobilize more neural resources to execute cognitive tasks of the same difficulty, potentially due to differences in cognitive resource availability or processing efficiency. This remains a theoretical interpretation rather than a confirmed mechanism.

## Author Contributions

**Yawei Li**: conceptualization, methodology, investigation, formal analysis, writing – original draft, writing – review and editing. **Tian Feng**: conceptualization, software, project administration, writing – review and editing, funding acquisition.

## Funding

This research was supported by the Science and Technology Project of Henan Province (grant no. 252102321129, to Yawei Li), the Young Teacher Training Program of Henan Higher Education Institutions (grant no. 2024GGJS162, to Tian Feng), and the Postdoctoral research funding of Henan Province (grant no. 348764, to Tian Feng).

## Ethics Statement

This study was approved by the Human Research Ethics Committee of Henan Sport University (approval no. 2024001). All procedures performed in studies involving human participants were in accordance with the ethical standards of the institutional and/or national research committee and with the 1964 Helsinki Declaration and its later amendments or comparable ethical standards.

## Consent

Written informed consent was obtained from all individual participants included in the study.

## Conflicts of Interest

The authors declare no conflicts of interest.

## Supporting information




**Supplementary Material**: brb371552‐sup‐0001‐SuppMat.xlsx

## Data Availability

The raw data that supports the findings of this study are available in the Supporting Information.
